# Trans Arterial Embolization of Spontaneous Intra-Abdominal Haemorrhage from Omental Lipoma

**DOI:** 10.1155/2018/2926143

**Published:** 2018-11-19

**Authors:** P. Tirukonda, S. Wu, J. Brar, K. S. Ng, S. Mirsadraee

**Affiliations:** ^1^Department of Radiology, Changi General Hospital, 2 Simei Street 3, 529889, Singapore; ^2^Department of Radiology, Royal Brompton Hospital, London, UK

## Abstract

We describe 3 cases of omental lipoma of whom 2 presented with symptomatic haemorrhage. Notably the haemorrhage in the 2 reported cases was from foregut arteries. Thorough knowledge of anatomy and embryology is critical in identifying the source of haemorrhage and differentiating this condition from other common causes of mesenteric haemorrhage. To the best of our knowledge, this is the first case series reporting this uncommon cause for abdominal haemorrhage. The successful management of this condition using superselective embolization is discussed. Clinicians need to exercise diligence and caution in omental lipomas presenting with spontaneous haemorrhage and this notion is exemplified in our reported cases.

## 1. Introduction

Omental lipoma is a benign tumor of mature adipose tissue [1]. Spontaneous symptomatic haemorrhage from omental lipoma is rare. The discussion of omental lipomas in the literature has been mostly limited to paediatric patients in case reports without any mention of omental haemorrhage in these patients [2]. However, there have been reports of cases with lipomas within the gastric or duodenal lumen resulting in gastrointestinal bleeding [3]. To our knowledge, we present the first case series of omental lipomas in middle-aged men, with active haemorrhage observed in 2 patients. Thorough knowledge of anatomy and embryology is critical in identifying the source of haemorrhage and differentiate this condition from other more common causes of mesenteric haemorrhage. The successful management of the condition using superselective embolization is discussed.

## 2. Case 1

A 50-year-old gentleman with hypertension, cholelithiasis, and previous left ureteric calculus had an episode of syncope and lower abdominal pain. On examination, he was tachycardic at 137 beats per minute and hypotensive at 68/53 mmHg. The patient was given intravenous 0.5 mg of adrenaline and started on a noradrenaline infusion during resuscitation. Ultrasound scan done in the Accident and Emergency Department showed a large amount of intra-abdominal free fluid.

**Figure 1:**
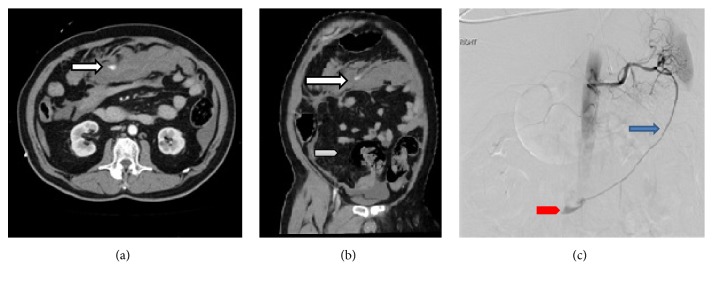
Case 1: Figures 1(a), 1(b), and 1(c). (a) Axial CT scan showing active contrast extravasation in the region of greater omentum. (b) Coronal MIP image showing actively bleeding aberrant artery arising from the splenic artery (arrow) and extending diagonally into the right iliac fossa, supplying the fat containing lesion (arrow head ). (c) Pre-embolization angiogram depicting the aberrant artery (blue arrow ). There is active contrast extravasation inferiorly (red arrow head ).

A CT mesenteric angiogram showed a large volume hemoperitoneum and active contrast extravasation in the region of the greater omentum (Figures 1(a) and 1(b)). The source of the haemorrhage was initially not discernible. However, after careful comparison with a previous CT scan done 3 years earlier, a long aberrant omental artery demonstrating a cockscrew pattern arising from the left gastro-epiploic artery was noted terminating in a large fat containing structure within the lower right abdomen.

A catheter angiogram was performed with a view to identify and embolize the bleeding vessel. Angiogram performed from the splenic artery confirmed the presence of an active haemorrhage from an aberrant artery which extended from the splenic hilum down to the right iliac fossa (Figure 1(c)). The artery terminated in a small cluster of abnormal cockscrew shaped vessels. Successful embolization of the artery was performed using coils and N-Butyl cyanoacrylate (glue). The patient remained haemodynamically stable and recovered well.

The CT scans were reviewed again in view of the abnormal vasculature in the right iliac fossa. It was noted that there actually was a large lipomatous lesion in the right iliac fossa. It had grown slightly in between the CT scans and was causing displacement of the small bowel loops. A MRI confirmed the presence of a fatty lesion with several abnormal internal vessels.

**Figure 2:**
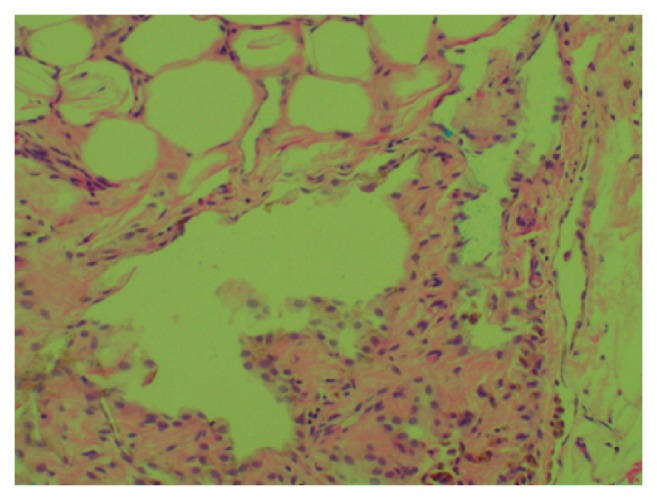
Case 1: Figure 2. Histology: Lipomatous tissue with focal fibrosis and old hemorrhage. There was no evidence of high grade sarcoma. Immunostains for CD31 and CD34 do not show evidence of abnormal vascular proliferation. FISH and MDM2 studies could not be performed due to lack of specimen.

Image guided biopsy of the lesion was performed. Pathological specimen of the fat-containing mass demonstrated lipomatous tissue with focal fibrosis and old haemorrhage (Figure 2). There was no evidence of high grade sarcoma. Immunostains for CD31 and CD34 were negative for abnormal vascular proliferation, reducing the likelihood of the mass being an angiomyolipoma. FISH and MDM2 studies could not be performed due to lack of specimen.

## 3. Case 2

A 57 year old gentleman with history of Child's C liver cirrhosis complicated by oesophageal varices and prior variceal bleed, alcohol dependence and multiple cardiovascular risk factors presented to our Emergency Department with abdominal pain and distension. A bedside abdominal tap revealed frank blood. He was hypotensive at presentation and his haemoglobin level dropped from a baseline of 10.5g/dL to 6.4g/dL. The coagulation factors were significantly deranged due to liver dysfunction.

**Figure 3:**
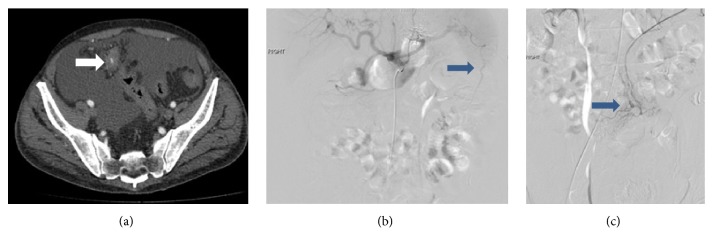
Case 2: Figures 3(a), 3(b), and 3(c). (a) Axial postcontrast CT showing contrast extravasation and surrounding haematoma within the lipomatous lesion (white arrow). Large amount of low density ascities is noted. (b & c): Selective catheterisation of the coeliac axis demonstrated an aberrant artery arising from the splenic artery crossing the midline to the right lower abdomen in the region of the acute haematoma terminating in abnormal cork screw vessels (blue arrows ).

A CT mesenteric angiogram was performed. It showed a 3.3 x 1.9cm haematoma in the sigmoid mesentery with a focus of contrast extravasation in the arterial phase that showed progressive pooling in the portal venous and delayed phases (Figure 3(a)). Subsequent catheter angiogram showed no contrast extravasation during selective catheterization of the superior and inferior mesenteric arteries. However, selective catheterization of the coeliac axis showed an aberrant vessel arising from the left gastro-epiploic artery and coursing obliquely to the right lower abdomen in the region of the haematoma (Figure 3(b)). Multiple abnormal vessels with a corkscrew appearance were also seen at the distal aspect of the aberrant artery (Figure 3(c)). Although no contrast extravasation was detected, decision was made for embolization in view of recent CT findings. Four 2mm fibered platinum coils were then deployed into this artery with good arterial stasis.

A repeat CT mesenteric angiogram performed 2 days later showed no contrast extravasation in the region of the haematoma. There were no further episodes of bleeding during this admission.

## 4. Case 3

A 71-year-old gentleman with multiple cardiovascular risk factors, known pancolonic diverticular disease and antral gastritis, was referred to our surgical service for per rectal bleeding. On examination, the patient was found to be pale, hypotensive (BP 80/50), and tachycardic (HR 108). Digital rectal examination revealed some stale melaena, although the patient reported passing moderate amounts of bright red blood in his stools intermittently.

His haemoglobin levels were 6.0g/dl on admission. He underwent fluid resuscitation and 4 units of packed red blood cell transfusion. Emergent esophagogastroduodenoscopy (OGD) and colonoscopy was performed which revealed areas of gastritis and pan diverticular disease but otherwise no sites of active ulcer or diverticular bleed.

**Figure 4:**
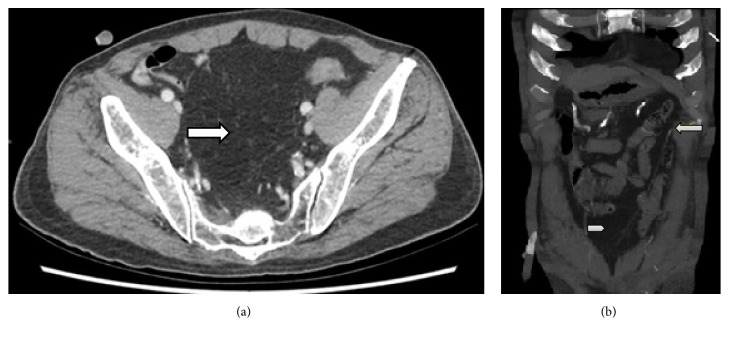
Case 3: Figures 4(a) and 4(b). (a) Axial CT image in the arterial phase showing a large fat-containing mass in the pelvis displacing bowel loops. No contrast extravasation is seen. (b) Coronal MIP CT images in the arterial phase showing aberrant vessel arising from the splenic artery (white arrow) and coursing obliquely from the left hypochondrium to the region of the pelvic mass (white arrowhead).

A CT mesenteric angiogram was performed which showed no active contrast extravasation in the arterial and portal venous phases. Dense material was noted within the ileum, probably from prior haemorrhage. There was an incidental finding of a fat-containing mass in the pelvis displacing the adjacent bowel loops (Figure 4(a)). There appeared to be an aberrant vessel arising from the left gastro-epiploic artery supplying the mass (Figure 4(b)). The CT scan showed no active contrast extravasation and, hence, no intervention was performed.

## 5. Discussion

We have described 3 cases of omental lipoma of whom 2 patients demonstrated acute intra-abdominal haemorrhage treated successfully by transarterial embolization. To our knowledge, there have been no previous reports of haemorrhage from omental lipoma in English literature.

Regardless of the location of the intra-abdominal lipomatous lesions, the arterial supply was from an aberrant branch arising from left gastro-epiploic artery close to the splenic hilum, which implies foregut embryological origin of these rare lesions (Figures 1(c), 3(b), and 4(b)). Anatomically this is likely to represent an aberrant omental artery. The conventional supply to the omentum is by three main arterial branches of the gastroepiploic artery namely, the right, middle, and left omental arteries. Thorough knowledge of embryology and previous imaging if available aids diagnosis and emergent management.

In the first case the source of the haemorrhage was unclear. Review of images did not reveal bleeding from the superior mesenteric artery. Review of previous CT performed 2 years ago, revealed an abnormal corkscrew vessel arising from the splenic hilum supplying a lipomatous lesion in the right iliac fossa.

In the second case, haematoma was seen in the region of the sigmoid mesentery, suggestive of a bleed from the inferior mesenteric artery. However, there was no active contrast extravasation during selective catheterization of the superior mesenteric and inferior mesenteric arteries. Selective cannulation of celiac axis demonstrated an aberrant vessel arising from the splenic hilum and coursing obliquely to the right lower abdomen in the region of the haematoma. The aberrant vessel was successfully embolized.

In the third case, no active contrast extravasation was seen in the arterial and portal venous phases. There was a large fat-containing mass in the pelvis resulting in mass effect. Once again there appeared to be similar pattern of aberrant branch arising from the splenic hilum supplying the mass.

The lipomatous lesions can easily be mistaken for normal mesenteric or omental fat. Careful examination can reveal mass effect such as displacement of bowel loops which was seen in our series (Figure 4(a)). Angiomyolipoma, liposarcoma, and lipoma with abnormal vasculature should always be considered in the differential for a fat-containing lesion [4].

A lipoma usually appears as a variably echogenic mass on ultrasound and may be encapsulated [5]. A liposarcoma can be distinguished from a lipoma by its display of heterogenous echotexture, large size or greater than minimal colour Doppler flow [5]. A lipoma may be distinguished from an angiomyolipoma by the former's lack of posterior acoustic shadowing on ultrasound [4].

While ultrasound allows for characterisation of a fat-containing mass, its anatomical location is best determined by CT or MRI [1]. On CT, an omental lipoma displays homogeneous fatty attenuation (< -60 Hounsfield units) [2]. It may occasionally display thin internal septa [6]. Presence of other internal components besides thin internal septa may suggest liposarcoma [6]. It can be distinguished from an omental liposarcoma by the latter's heterogeneous attenuation, calcification and presence of local invasion [7].

MRI is highly sensitive in characterizing typical features associated with well differentiated liposarcoma and highly specific in diagnosing simple lipoma [6]. With MRI pulse sequences, a lipoma demonstrates homogeneous signal intensity similar to that of fat, i.e., high T1 signal and intermediate T2 signal [8]. It saturates on fat saturated T1 and T2 sequences. Thin fibrous septa of low T1-weighted and T2-weighted signal intensity may be seen within the mass [4]. However, Low grade liposarcomas may not be distinguished from benign lipomas based on imaging alone. Therefore, histological confirmation may be necessary. It is noteworthy that imaging features of lipomas can be similar to well-differentiated liposarcomas. Presence of imaging features such as thick enhancing septa, presence of nodular and/or globular, or nonadipose mass like areas and decreased percentage of fat within the lesion are suggestive of malignancy [7].

A lipoma cannot be distinguished from an angiomyolipoma on CT and MRI, as the latter will also display imaging features of macroscopic fat and rarely calcification. Histological differentiation is required [4]. Lack of staining with HMB45, CD31 and CD34 reduces the likelihood of the mass being an angiomyolipoma [9].

Pathological specimen of the fat-containing mass in the first patient displayed lipomatous tissue with focal fibrosis and old haemorrhage. Pathology report noted that there was no evidence of high grade sarcoma. Immunostains for CD31 and CD34 were negative for abnormal vascular proliferation, thereby ruling out angiomyolipoma.

Definitive management of a symptomatic lipoma includes surgical resection [10]. There is a postsurgical recurrence rate of less than 5% [10].

## 6. Conclusion

An omental lipoma is a rare benign tumour. In this case series, we have described 3 patients with omental lipomas, of whom two patients presented with active haemorrhage. Thorough knowledge of anatomy and embryology, as well as previous imaging, can aid diagnosis. Superselective transarterial embolization is safe, minimally invasive, and effective and hence should be offered as the first line treatment approach in the management of bleeding omental lipoma.
